# The annual carnival in Guadeloupe (French West Indies) is associated with an increase in the number of conceptions and subsequent births nine months later: 2000 – 2011

**DOI:** 10.1371/journal.pone.0173102

**Published:** 2017-03-02

**Authors:** Philippe Kadhel, Nathalie Costet, Teddy Toto, Eustase Janky, Luc Multigner

**Affiliations:** 1 Centre Hospitalier et Universitaire de Pointe-à-Pitre/Abymes, Pôle Parent-Enfant, Service de Gynécologie et Obstétrique, Pointe-à-Pitre, Guadeloupe, France; 2 Université des Antilles, Campus de Fouillole, Pointe-à-Pitre, Guadeloupe, France; 3 Institut national de la santé et de la Recherche médicale (Inserm) U1085 – IRSET, Rennes, France; 4 Université de Rennes 1, Rennes, France; TNO, NETHERLANDS

## Abstract

The seasonal patterns of conceptions and births differ between geographic areas. Several potential determinants of this variation have been identified, including biological, environmental and behavioral elements, but festive events are rarely mentioned. We investigated the possible association between the carnival and seasonal fertility variations in the French West Indies. We ran a retrospective registry-based study. The data were extracted from the registry of all births on Guadeloupe between 2000 and 2011 (*n* = 74,412), and from the Maternity Birth Register of the University Hospital, for all pregnancies of at least 14 completed weeks of gestation (observable conceptions) with an outcome recorded between 2007 and 2010 (*n* = 8,425). We compared data during and outside the carnival period for each year, including 2009, when there was no carnival due to a 44-day general strike. In all years other than 2009, the weekly number of births was higher for pregnancies initiated during the carnival period than for pregnancies initiated at other times, and the weekly number of observable conceptions was higher during the carnival period than at other times. Our findings support the hypothesis that carnivals in the French West Indies are associated with an increase in the number of conceptions and subsequent births.

## Introduction

Conception and birth frequencies have been shown to display seasonal variations at almost all sites studied worldwide [1]. Biological, nutritional, economic and sociodemographic factors, including sperm quality, ovarian function, age, education, agricultural cycles, climatic conditions, and behavioral elements affecting the frequency of intercourse, such as seasons in which marriages are more frequent, festivities and holidays, have all been identified as potential determinants of the seasonality of conceptions and births [2–9]. Depending on the factors involved, seasonal conception and birth patterns may differ between geographic areas and over time [5].

Previous studies have investigated the contribution of secular or religious festivities to birth seasonality. New Year's Day (January 1), the first day of the year in the modern Gregorian calendar and a public holiday in many countries, has been associated with an increase in the number of births in late September in France [7, 10]. Similarly, in England and Wales, a small peak in the number of births has been reported in September, corresponding to conception during the Christmas holidays [11]. The period following the annual Hajj pilgrimage to Mecca has been associated with an increase in the number of births nine months later among Muslims living in Southern Israel [12]. An increase in the number of births was also reported nine months after the 2010 FIFA World Cup in South Africa [13].

The carnival is an annual festive period traditionally held in areas with a strong Roman Catholic tradition. In Guadeloupe (French West Indies), the carnival starts in January and lasts for between four and 10 weeks. Local obstetricians suspect that the annual carnival is associated with an increase in the number of births nine months later, but no data have ever been published to support this assertion. We investigated the possible effects of the carnival period in Guadeloupe on the number of conceptions and subsequent births, for every year from 2000 through 2011. This period included 2009, a year in which the carnival festivities were canceled due to a general strike.

## Materials and methods

This study took place in Guadeloupe (French West Indies), a tropical archipelago in the Caribbean covering an area of 1,628 km^2^, with 402,000 inhabitants (in 2013). The population is mostly of African origin, descended from slaves who arrived from West Africa in the 17th, 18th and early 19th centuries.

We used two different data sources: the Guadeloupean birth register, covering the entire territory and recording only administrative information, and the birth register from the maternity unit of Guadeloupe University Hospital, which provided individual information about gestational age.

Annual and weekly numbers of births from January 1 2000 through December 31 2011 collected by the Guadeloupean Birth Register were provided by the *Direction des Actions de Solidarité Départementale*. The French West Indies are an integral part of France, and 100% of civilian births are officially registered. Weekly numbers of births were not available for 2006, due to a loss of data from the computer system. Weeks were defined according to the Gregorian calendar, beginning on Monday and ending the following Sunday. Births were considered to be any product of conception weighing at least 500 g or having completed 22 full weeks of gestation [14]. The registration of births is obligatory for all children born in Guadeloupe, regardless of the nationality of the mother. We calculated a period for each year, in weeks, corresponding to births resulting from conceptions occurring during the previous carnival. A normal modal duration in days of gestation is commonly used in specific populations, mostly of Caucasian descent, and consequently may not be adapted to populations of African descent [15]. We assumed that all pregnancies were of normal duration, defined as between 37 and 42 complete weeks of gestation, which is considered normal for all populations [14]. As ovulation occurs two weeks after the first day of normal menstruation, the normal time from conception to delivery is thus between 35 and 40 complete weeks. We then defined a birth period for “carnival pregnancies” for a given year, based on pregnancies of normal length for which conception occurred during the carnival period. These birth periods were defined as extending from 35 weeks after the first day of the carnival to 40 weeks after the last day of the carnival.

Pregnancy outcomes and gestational age for all pregnancies of at least 14 completed weeks of gestation seen at the maternity unit of Guadeloupe University Hospital from January 1 2007 through October 31 2011 were provided by the hospital (*Centre Hospitalier Universitaire Pointe à Pitre/Abymes*). During this period, 34 to 40% of all births occurring annually in Guadeloupe occurred at this center. We calculated the date of conception from individual information about gestational age (in days) for all pregnancies of at least 14 completed weeks of gestation (observable conceptions) with an outcome recorded at Guadeloupe University Hospital. This approach thus excluded miscarriages and abortions occurring before 14 completed weeks of gestation. We subtracted the gestational age, in days, from the date of the recorded pregnancy outcome, to obtain the date of conception. We retained all observable conceptions occurring from January 1 2007 through December 31 2010.

Population data for each year from 2000 through 2011 were obtained from the French National Institute for Statistics and Economic Studies (*Institut national de la statistique et des études économiques;* https://www.insee.fr/*)*. Weather conditions have frequently been reported to be associated with birth seasonality [6]. Meteorological reference parameters (mean temperature, precipitation and hours of sunshine) were recorded daily, from January 1 2000 through December 31 2011, at Raizet Airport weather station (no. 97101015), and were provided by Météo-France Services.

### Statistical analysis

For each year, we compared the weekly numbers of births occurring during and outside the birth period defined for carnival pregnancies and the weekly number of observable conceptions occurring during and outside the carnival period. We also compared gestational age of observable conceptions occurring during and outside the carnival period. The weekly numbers of births and conceptions, and gestational age were not normally distributed. We therefore used nonparametric Mann-Whitney tests to compare the groups. In addition, we used regression models to compare the weekly numbers of births and conceptions during and outside the birth period defined for carnival pregnancies and the carnival period, respectively. Negative binomial regression models were used to handle over-dispersed data (variance greater than the sample mean). We took the unusual characteristics of 2009 into account by using a composite variable (interaction term) combining the year and the carnival period indicator (during *vs*. outside the carnival period). We used two different models: Model 1: “weeks outside the carnival period for all years” was compared with “weeks during the carnival in all years except 2009” and “weeks during the carnival period in 2009”. Model 2: “weeks outside the carnival period in all years except 2009” was compared with “weeks outside the carnival period in 2009”, “weeks during the carnival period in 2009”, and “weeks during the carnival in all years except 2009”. The response variable was the weekly number of births or conceptions. Results are expressed as the percentage of births or conceptions ([^ebβ^ – 1] x 100). Both models were adjusted for meteorological parameters (mean weekly temperature, precipitation and hours of sunshine). Mean daily values for meteorological parameters during the carnival period, from 2000 to 2011 were compared in Student’s *t* tests. All tests were two-tailed and values of *P* ≤0.05 were considered statistically significant. Statistical analysis was performed with SAS ^®^ software, version 9.3.

## Results

In Guadeloupe, there were 74,105 births in total between January 1 2000 and December 31 2011. Annual birth rates decreased steadily over this period, from 17.6 per thousand in 2000 to 13.1 per thousand in 2011 (Table 1).

**Table 1:** Weekly number of births occurring during and outside the birth period for carnival pregnancies from January 1 2000 to December 31 2011 in Guadeloupe (French West Indies).

Year	Births	Birth rate	Births from pregnancies initiated outside the carnival period		Births from pregnancies initiated during the carnival period		*p* value ^a^
	*n*	per 1,000	*n*	Weekly births mean (range)	*n*	Weekly births mean (range)	
2000	6,836	17.6	4,680	126.5 (94–154)	2,156	131.5 (125–173)	<0.001
2001	6,749	17.3	4,855	124.5 (89–161)	1,894	145.7 (117–167)	<0.001
2002	6,178	15.7	4,714	115 (89–146)	1,464	133.1 (104–172)	0.005
2003	6,241	15.8	4,459	117.3 (87–160)	1,782	127.3 (113–141)	0.008
2004	6,426	16.6	4,595	117.8 (90–152)	1,831	140.8 (117–165)	<0.001
2005	6,669	16.7	5,086	124 (82–149)	1,583	143.9 (129–163)	<0.001
2006	6,430	16.0	- ^b^	- ^b^	- ^b^	- ^b^	-
2007	6,226	15.5	4,612	115.3 (90–147)	1,614	134.5 (115–150)	<0.001
2008	5,900	14.7	4,685	111.5 (92–132)	1,215	121.5 (101–142)	0.05
2009	5,683	14.5	4,168	106.9 (68–131)	1,515	116.5 (98–140)	0.11
2010	5,468	13.6	4,117	102.9 (82–128)	1,351	112.6 (86–142)	0.05
2011	5,299	13.1	3609	97.5 (76–122)	1,690	112.7 (86–144)	0.007
All years ^c^	67,675	-	49,580	114.5 (68–161)	18,095	130.2 (86–173)	<0.001

^a^ for differences in the continuous distribution of weekly number of births (Mann-Whitney test).

^b^ data concerning the weekly numbers of births were not available.

^c^ except 2006.

For all years, 2000 through 2011, the overall weekly number of births was significantly higher during the birth periods for carnival pregnancies than at other times of year (Table 1). An analysis of the data by year revealed that the weekly number of births was significantly higher during the birth periods for carnival pregnancies than at other times of year, for each year except 2009, the year in which the carnival was canceled (Table 1). Negative binomial regression analysis showed that the number of births during the birth period for carnival pregnancies was significantly higher than that for other periods of the year, in all years except 2009 (15%, 95% confidence interval [CI]: 11 to 18% in Model 1; 14%, 95%CI: 11 to 17% in Model 2) whereas no such difference was observed in 2009 (1%, 95% CI: -6 to 9% in Model 1; 1%, 95% CI: -7 to 9% in Model 2) (S1 Table).

**Fig 1:**
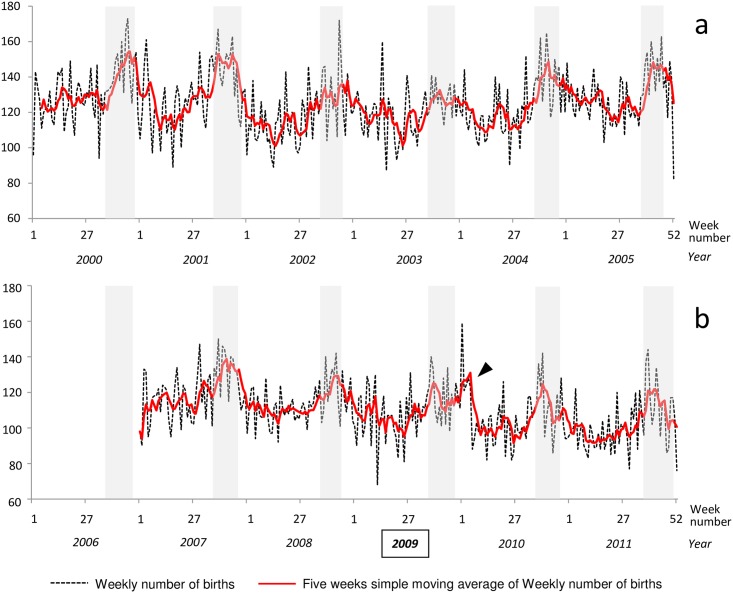
Weekly number of births in Guadeloupe (French West Indies) from January 1 2000 through December 31, 2011. a: January 1 2000 to December 31, 2005. b: January 1 2007 to December 31, 2011.

The weekly number of births and the unweighted five-week moving average of the weekly number of births are shown in Fig 1. If birth periods for carnival pregnancies were plotted as shaded areas, they largely overlapped the period of the year during which the weekly number of births was highest, in all years except 2009, in which the weekly number of births was highest in the month following the theoretical birth period for carnival pregnancies (arrow in Fig 1).

Shaded areas: periods of births for pregnancies initiated during the carnival period of the same year. Weekly numbers of births were not available for 2006.

Arrow: peak of weekly numbers of births that follows the theoretical period of birth for 2009 carnival pregnancies.

From January 1 2007 through December 31 2010, we estimated that there were 8,425 conceptions resulting in a pregnancy continuing to at least 14 complete weeks of gestation recorded at the maternity unit of Guadeloupe University Hospital. During this period, the weekly number of conceptions was significantly higher during the carnival period than outside this period (Table 2). An analysis of the data by year revealed that the weekly number of conceptions during the carnival period was significantly higher or almost so than that outside this period, for each year except 2009 (Table 2). Negative binomial regression analysis showed that the number of conceptions was higher during the carnival period than at other times of the year, although not significantly so, in all years except 2009 (8%, 95% CI: -2 to 18% in Model 1; 8%, 95% CI: -1 to 19% in Model 2; data for 2009: -6%, 95% CI: -18 to 8% in Model 1; -5%, 95% CI: -17 to 8% in Model 2) (S2 Table).

**Table 2:** Weekly number of conceptions occurring during and outside the carnival period, from January 1 2007 to December 31 2010 recorded at the University Hospital in Guadeloupe (French West Indies).

Year	Conceptions	Conceptions outside the carnival period		Conceptions during the carnival period		*p* value ^a^
	*n*	*n*	Weekly conceptions mean (range)	*n*	Weekly conceptions mean (range)	
2007	2,144	1,799	40.0 (26–60)	345	49.3 (42–64)	0.003
2008	2,018	1,794	38.2 (24–51)	224	44.8 (34–56)	0.06
2009	2,166	1,832	40.7 (25–63)	334	41.8 (31–57)	0.70
2010	2,142	1,812	40.3 (19–67)	330	47.1 (38–56)	0.08
All years	8,470	7,237	39.8 (19–67)	1,233	45.7 (31–64)	<0.001

^a^ for differences in the continuous distribution of weekly number of conceptions (Mann-Whitney test).

**Fig 2:**
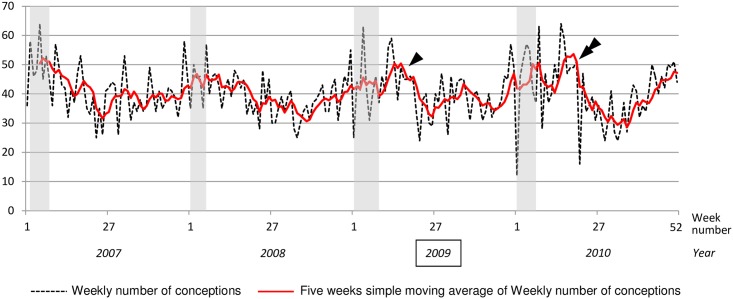
Weekly number of conceptions in Guadeloupe (French West Indies) from January 1 2007 through December 31, 2010. Shaded areas: periods of conceptions initiated during the carnival period of the same year. Arrow: peak of weekly numbers of conceptions that follows the period of canceled 2009 carnival. Double arrow: peak of weekly numbers of conceptions that follows the period of 2010 carnival.

The weekly number of observable conceptions and the unweighted five-week moving average of the weekly number of conceptions are shown in Fig 2. If the conception periods for carnival pregnancies were plotted as shaded areas, they overlapped the period of the year during which the weekly number of conceptions was highest, in all years except 2009. In 2009, the weekly number of conceptions was highest after the cancelled carnival period. In 2010, the carnival period coincided with a high weekly number of conceptions, but a second peak in the number of conceptions was also observed a few weeks later (double arrow in Fig 2).

There was no significant difference in gestational age between births resulting from conceptions during the carnival and non-carnival periods for 2007 and 2008 (in which the carnival took place) and 2009 (no carnival) (p values: 0.27, 0.11, and 0.69, respectively). However, we observed a significant difference for 2010 (in which the carnival took place)(p value < 0.001; mean gestational age of 250 days for the carnival period and 257 days for the non-carnival period). Mean daily temperature, precipitation and hours of sunshine for the carnival period in 2009 did not differ from those in the other years from 2000 through 2011 (see Supporting Information, S3 Table).

## Discussion

We show here that the number of planned or accidental but accepted observable conceptions increases during the carnival period and that the number of births following conceptions during the carnival period is higher than that for conceptions at other times of year in Guadeloupe. These findings were observed for all years investigated except 2009, the year in which the carnival was canceled due to a general strike.

This study was carried out in a French territory in which birth registration is compulsory. Moreover, the insular nature of Guadeloupe and the status of the surrounding islands as independent English-speaking states limited the number of births occurring elsewhere after conception in Guadeloupe and the number of births occurring in Guadeloupe after conception elsewhere. We therefore consider our estimate of the numbers of births to be accurate.

When determining the number of births resulting from observable conceptions in a given period, a normal duration of gestation, such as 266 days, for example, is generally used [7, 16]. This value corresponds to the modal duration of gestation in specific populations, mostly of Caucasian descent, and may therefore not be representative of our population of individuals mostly of African descent [15]. We therefore considered a wide range of durations, between 37 and 42 weeks of gestation, to be normal, as this range is generally considered normal for all populations [14]. We recognize that this approach, facilitated by the accessibility of the birth registry, could be called into question because some births occurred after pregnancies lasting shorter or longer than this duration defined as “normal”. We tried to mitigate the effects of this limitation by also collecting information about the gestational age of the fetus, in days, for all pregnancies continuing for at least 14 weeks of gestation for which an outcome was recorded at the maternity unit of Guadeloupe University Hospital during a period of four consecutive years, including 2009, the year in which the carnival was canceled. This made it possible to determine the date of conception for each of these pregnancies. Similar conclusions were drawn from the results obtained with these two approaches.

This study generated only retrospective and descriptive data. We cannot, therefore, infer causality in the observed association between the carnival period and the increase in the number of observable conceptions and subsequent births. However, we can put forward non-mutually exclusive hypotheses about this association. At first glance, it could be assumed that the carnival is a period of unbridled pleasure involving fun, alcohol and recreational drug abuse, leading to unrestricted and unprotected sex. Such permissive behavior would lead to an increase in the risk of individuals contracting sexually transmitted infections. However, a Brazilian study reported that the carnival had no influence on the incidence of sexually transmitted diseases [17]. We also feel that this vision is too simplistic and that it does not correspond to the reality observed in Guadeloupe. The carnival period in Guadeloupe consists of normal working days interspersed with public holidays. The festivities are characterized by numerous weekend parades in the various towns, organized by official carnival associations and the city halls, daily unofficial street parades in a friendly atmosphere, and public and private parties. People of all social categories and ages, including kindergarten children, scholars and retirement home residents, participate in these events. Moreover, even if carnival festivities did lead to more sexual activity, the population of Guadeloupe has free access to contraception and legal abortion, which would probably restrict increases in unplanned or unwanted pregnancies. Also, we cannot exclude that carnival-related factors could modify gestational age. We did not observed consistent differences in gestational age between births resulting from conceptions during the carnival and non-carnival periods. It therefore seems unlikely that these factors are responsible for the seasonal variations of birth rate observed.

To our knowledge, there is no popular tradition recommending conception during the January-February carnival period. Births resulting from observable conceptions during this period are expected in October/November and we are aware of no particular factor that might influence decisions to conceive a child such that it would be born at this specific time in the year. Marriage now has less influence over conception period, but the traditional season for weddings in Guadeloupe is the July and August vacation period. Nutritional and energetic factors, frequently related to agricultural cycles, should also be taken into account, because they may influence ovarian function [18]. However, there is no particular reason to suspect a role for such factors here, because the Guadeloupean population has access to adequate supplies of local and imported food resources at all times of year, with no effect of agricultural season. Weather conditions, such as air temperature and the number of hours of sunshine, are known to influence birth seasonality [6]. However, Guadeloupe has a tropical climate displaying little variation in temperature (mean daily temperature fluctuates between 24°C and 27°C) and a constant day length (about 12.5 hours of daylight) throughout the year. It has been suggested that heavy rainfall may increase the frequency of intercourse, as couples spend more of their leisure time indoors [19]. However, the carnival period in the French West Indies takes place during the dry season.

Seasonal patterns in birth rates may not persist over time. The difference in the weekly numbers of births from pregnancies initiated during and outside the carnival period (except for 2009) remained significant throughout the study, but seemed to be decreasing over time. This may reflect the trend towards a decrease in annual birth rates, affecting pregnancies beginning during and outside the carnival period equally (Table 1), or undetermined changes in sociocultural, behavioral and/or economic factors in Guadeloupe.

No increase in the number of observable conceptions or in the number of births from pregnancies initiated during the theoretical carnival period was observed in 2009, the year in which the carnival festivities were canceled. The weather conditions during this period in 2009 were no different from those of the carnival periods in other years. This suggests that the festivities associated with the carnival, rather than the timing of the annual carnival period, underlie the increase in conception and subsequent birth rates.

The cancelation of carnival festivities in 2009 was an unusual situation, which had not occurred for several decades at least in Guadeloupe. This cancelation was due to a general strike and street demonstrations in response to increases in the price of basic commodities. As a consequence, stores, gas stations and various services, including those relating to education, transportation and sanitation, shut down for 44 consecutive days. We cannot exclude the possibility that stress factors relating to the social crisis affected the numbers of conceptions and subsequent births [20]. Traumatic collective events, such as earthquakes, floods, storms, and city bombings have been associated with variations in reproductive outcomes, such as decreases in the frequency of intercourse [21], increases in menstrual disorders [21], decreases in sperm motility [22], decreases in the sex ratio of the children conceived [23], and changes in the number of births, with both increases and decreases reported [24–26].

In this context, it is not clear how the increase in the weekly numbers of births for carnival pregnancies or observable conceptions a month after the theoretical period for carnival in 2009 (arrows in Figs 1 and 2) should be interpreted. This rebound effect is probably interpretable only in light of the reasons for the decrease observed during the cancellation of the carnival festivities in 2009. Further analyses, including additional epidemiologic data (marriage, divorce, abortion, depression or other psychological problems), as used in the case of the Oklahoma City bombing and Hurricane Hugo analyses [24, 25], are required to explore this aspect more thoroughly. More surprising is the unexplained persistence of a rebound in conceptions recorded at the University Hospital in 2010 (double-arrow in Fig 2) but not in births registered in the Guadeloupean birth registry.

We believe that knowledge about the seasonality of conception and birth and its determinants is not merely anecdotal. An understanding of such seasonal variations is of potential importance for public health policy. The periods in which conception frequency is highest could be chosen for focused interventions against specific pregnancy risks, such as campaigns for the prevention of sexually transmitted diseases or alcohol consumption during pregnancy. Such knowledge would help health institutions to target and time their delivery and postnatal care interventions more appropriately. Also, it may be important for research on the impact of the seasonality of conception and birth rates on subsequent medical conditions, in both children and adults. The development of a number of medical conditions, including congenital abnormalities, schizophrenia, multiple sclerosis and other neurological disorders, type I diabetes, and cancers, and the dynamics of acute immunizing infections have been shown to be influenced by the season of conception or birth [27–33]. Adult life expectancy has also been shown to be related to month of birth [34]. Finally, because the sex ratio has been suggested to be a sentinel environmental health indicator [35], it may also be informative to explore the influence of festive periods on the sex-ratio.

## Conclusion

In summary, we show here that a festive period of several weeks, such as the carnival in Guadeloupe, can influence birth seasonality. Our study confirms the idea raised by other studies that festive periods may influence human fertility. However, our findings require confirmation in other populations. Sociocultural factors differ between countries and may therefore have different effects on birth seasonality. The interactions between biological and social variables may also differ between times and places.

## Supporting information

S1 Table Parameter Estimates for births in Guadeloupe (French West Indies): 2000–2011. ^**a**^ Estimates and standard errors from a binomial negative regression model, adjusted to mean weekly temperature, mean weekly precipitation and mean weekly hours of sunshine.
(DOCX)

S2 Table Parameter Estimates for conceptions in the University Hospital of Guadeloupe (French West Indies): 2007–2010. ^**a**^ Estimates and standard errors from a binomial negative regression model, adjusted to mean weekly temperature, mean weekly precipitation and mean weekly hours of sunshine.
(DOCX)

S3 Table Daily meteorological parameters during the carnival period in Guadeloupe (French West Indies): 2000–2011. ^a^ Data were provided by Météo-France services. ^b^ For differences in means (Student’s *t* test).
(DOC)
