# Supplementary material for: The annual carnival in Guadeloupe (French West Indies) is associated with an increase in the number of conceptions and subsequent births nine months later: 2000 – 2011
Source: PLoS One. 2017 Mar 2;12(3):e0173102. doi: 10.1371/journal.pone.0173102 (PMC5333860; doi:10.1371/journal.pone.0173102)
Supplement: S1 Table — a Estimates and standard errors from a binomial negative regression model, adjusted to mean weekly temperature, mean weekly precipitation and mean weekly hours of sunshine. (DOCX) [file pone.0173102.s001.docx]

**Supplemental Table S1. Parameter Estimates for births in Guadeloupe (French West Indies): 2000-2011**

**Model 1 ^a^**

| **Parameters** | **β** | **Std. Error** | **95% CI** | | **Hypothesis test** | | **Expβ** | **95% CI for Expβ** | |
| --- | --- | --- | --- | --- | --- | --- | --- | --- | --- |
|  |  |  | **Lower** | **Upper** | **Wald Chi-Square** | ***P* value** |  | **Lower** | **Upper** |
| Weeks outside the carnival period for all years whatever the year | Ref (0) |  |  |  |  |  | 1.0 |  |  |
| Weeks during the carnival period in all years except 2009 | 0.137 | 0.014 | 0.1087 | 0.165 | 90.76 | <0.001 | 1.147 | 1.115 | 1.180 |
| Weeks during the carnival period in 2009 | 0.012 | 0.039 | -0.0641 | 0.088 | 0.09 | 0.760 | 1.012 | 0.938 | 1.092 |

**Model 2 ^a^**

| **Parameters** | **β** | **Std. Error** | **95% CI** | | **Hypothesis test** | | **Expβ** | **95% CI for Expβ** | |
| --- | --- | --- | --- | --- | --- | --- | --- | --- | --- |
|  |  |  | **Lower** | **Upper** | **Wald Chi-Square** | ***P* value** |  | **Lower** | **Lower** |
| Weeks outside the carnival period in all years except 2009 | Ref (0) |  |  |  |  |  | 1.0 |  |  |
| Weeks outside the carnival period in 2009 | -0.059 | 0.023 | -0.105 | -0.014 | 6.51 | 0.011 | 0.943 | 0.901 | 0.986 |
| Weeks during the carnival period in 2009 | 0.007 | 0.039 | -0.069 | 0.083 | 0.03 | 0.857 | 1.007 | 0.934 | 1.086 |
| Weeks during the carnival period in all years except 2009 | 0.132 | 0.014 | 0.104 | 0.161 | 84.34 | <0.001 | 1.142 | 1.110 | 1.174 |

**^a^** Estimates and standard errors from a binomial negative regression model, adjusted to mean weekly temperature, mean weekly precipitation and mean weekly hours of sunshine
