# Supplementary material for: The annual carnival in Guadeloupe (French West Indies) is associated with an increase in the number of conceptions and subsequent births nine months later: 2000 – 2011
Source: PLoS One. 2017 Mar 2;12(3):e0173102. doi: 10.1371/journal.pone.0173102 (PMC5333860; doi:10.1371/journal.pone.0173102)
Supplement: S2 Table — a Estimates and standard errors from a binomial negative regression model, adjusted to mean weekly temperature, mean weekly precipitation and mean weekly hours of sunshine. (DOCX) [file pone.0173102.s002.docx]

**Supplemental Table S2. Parameter Estimates for conceptions in the University Hospital of Guadeloupe (French West Indies): 2007-2010**

**Model 1 ^a^**

| **Parameters** | **β** | **Std. Error** | **95% CI** | | **Hypothesis test** | | **Expβ** | **95% CI for Expβ** | |
| --- | --- | --- | --- | --- | --- | --- | --- | --- | --- |
|  |  |  | **Lower** | **Upper** | **Wald Chi-Square** | ***P* value** |  | **Lower** | **Upper** |
| Weeks outside the carnival period for all years whatever the year | Ref (0) |  |  |  |  |  | 1.0 |  |  |
| Weeks during the carnival period in all years except 2009 | 0.074 | 0.046 | -0.017 | 0.165 | 2.51 | 0.113 | 1.076 | 0.983 | 1.179 |
| Weeks during the carnival period in 2009 | -0.06 | 0.068 | -0.193 | 0.073 | 0.78 | 0.376 | 0.942 | 0.825 | 1.076 |

**Model 2 ^a^**

| **Parameters** | **β** | **Std. Error** | **95% CI** | | **Hypothesis test** | | **Expβ** | **95% CI for Expβ** | |
| --- | --- | --- | --- | --- | --- | --- | --- | --- | --- |
|  |  |  | **Lower** | **Upper** | **Wald Chi-Square** | ***P* value** |  | **Lower** | **Upper** |
| Weeks outside the carnival period in all years except 2009 | Ref (0) |  |  |  |  |  | 1.0 |  |  |
| Weeks outside the carnival period in 2009 | 0.021 | 0.031 | -0.0406 | 0.082 | 0.44 | 0.507 | 1.021 | 0.960 | 1.085 |
| Weeks during the carnival period in 2009 | -0.054 | 0.069 | -0.1878 | 0.080 | 0.62 | 0.431 | 0.948 | 0.829 | 1.083 |
| Weeks during the carnival period in all years except 2009 | 0.080 | 0.047 | -0.0129 | 0.173 | 2.85 | 0.092 | 1.083 | 0.987 | 1.189 |

**^a^** Estimates and standard errors from a binomial negative regression model, adjusted to mean weekly temperature, mean weekly precipitation and mean weekly hours of sunshine
