# Supplementary material for: The annual carnival in Guadeloupe (French West Indies) is associated with an increase in the number of conceptions and subsequent births nine months later: 2000 – 2011
Source: PLoS One. 2017 Mar 2;12(3):e0173102. doi: 10.1371/journal.pone.0173102 (PMC5333860; doi:10.1371/journal.pone.0173102)
Supplement: S3 Table — a Data were provided by Météo-France services. b For differences in means (Student’s t test). (DOC) [file pone.0173102.s003.doc]

**Supplemental Table S3. Daily meteorological parameters during the carnival period in Guadeloupe (French West Indies): 2000-2011**

| **Parameters  ^a^** | **Years** | | | | | ***P* value ^b^** |
| --- | --- | --- | --- | --- | --- | --- |
|  | 2009 | |  | 2000 to 2011 except 2009 | |  |
|  | Mean | (range) |  | Mean | (range) |  |
| Mean temperature (°C) | 24.5 | (23.0-25.9) |  | 24.4 | (21.5-26.6) | 0.68 |
| Precipitation (mm) | 2.5 | (0.0-10.4) |  | 2.9 | (0.0-107.5) | 0.67 |
| Sunshine duration (min) | 394 | (159-595) |  | 372 | (0.0-683) | 0.24 |

^a^ Data were provided by Météo-France© services.

^b^ For differences in means (Student’s *t* test).
